# Supplementary material for: Transcript assembly and annotations: Bias and adjustment
Source: PLoS Comput Biol. 2023 Dec 21;19(12):e1011734. doi: 10.1371/journal.pcbi.1011734 (PMC10769104; doi:10.1371/journal.pcbi.1011734)
Supplement: S1 Text — (PDF) [file pcbi.1011734.s001.pdf]

Supplementary Materials for
“Transcript Assembly and Annotations: Bias and Adjustment”

Qimin Zhang[1] and Mingfu Shao[1,2,*]

[1]Department of Computer Science and Engineering, School of Electrical Engineering and Computer Science, The Pennsylvania State University, University Park, Pennsylvania, United States of America
[2]Huck Institutes of the Life Sciences, The Pennsylvania State University, University Park, Pennsylvania, United States of America
[*]Corresponding author: Mingfu Shao, mxs2589@psu.edu

December 11, 2023

List of Figures

A The impact of intron retention filtering on assembly accuracy with RefSeq and Ensembl annotations from GRCh38 genome build on EN10 dataset. 2
B The impact of intron retention filtering on assembly accuracy with RefSeq and Ensembl annotations from GRCh38 genome build on HS7 dataset. 3
C The impact of intron retention filtering on assembly accuracy with RefSeq and Ensembl annotations from T2T-CHM13 genome build on EN10 dataset. 4
D The impact of intron retention filtering on assembly accuracy with RefSeq and Ensembl annotations from T2T-CHM13 genome build on HS7 dataset. 5
E Performance evaluation of irtool with varying length ratio parameter. 6
F Performance evaluation of irtool with varying coverage ratio parameter. 7

List of Tables

A Comparison of assembly accuracy of StringTie2 and Scallop2-ft on different annotations. . . 8
B Comparison of assembly accuracy of StringTie2-ft and Scallop2-ft on different annotations. . 9
C The impact of filtering partial/entire intron retention on assembly accuracy. 10
D Comparison of assembly accuracy of four methods on RefSeq and Ensembl annotations. . . 11
E Comparison of assembly accuracy of four methods evaluated using union and intersection RefSeq and Ensembl annotations. 12

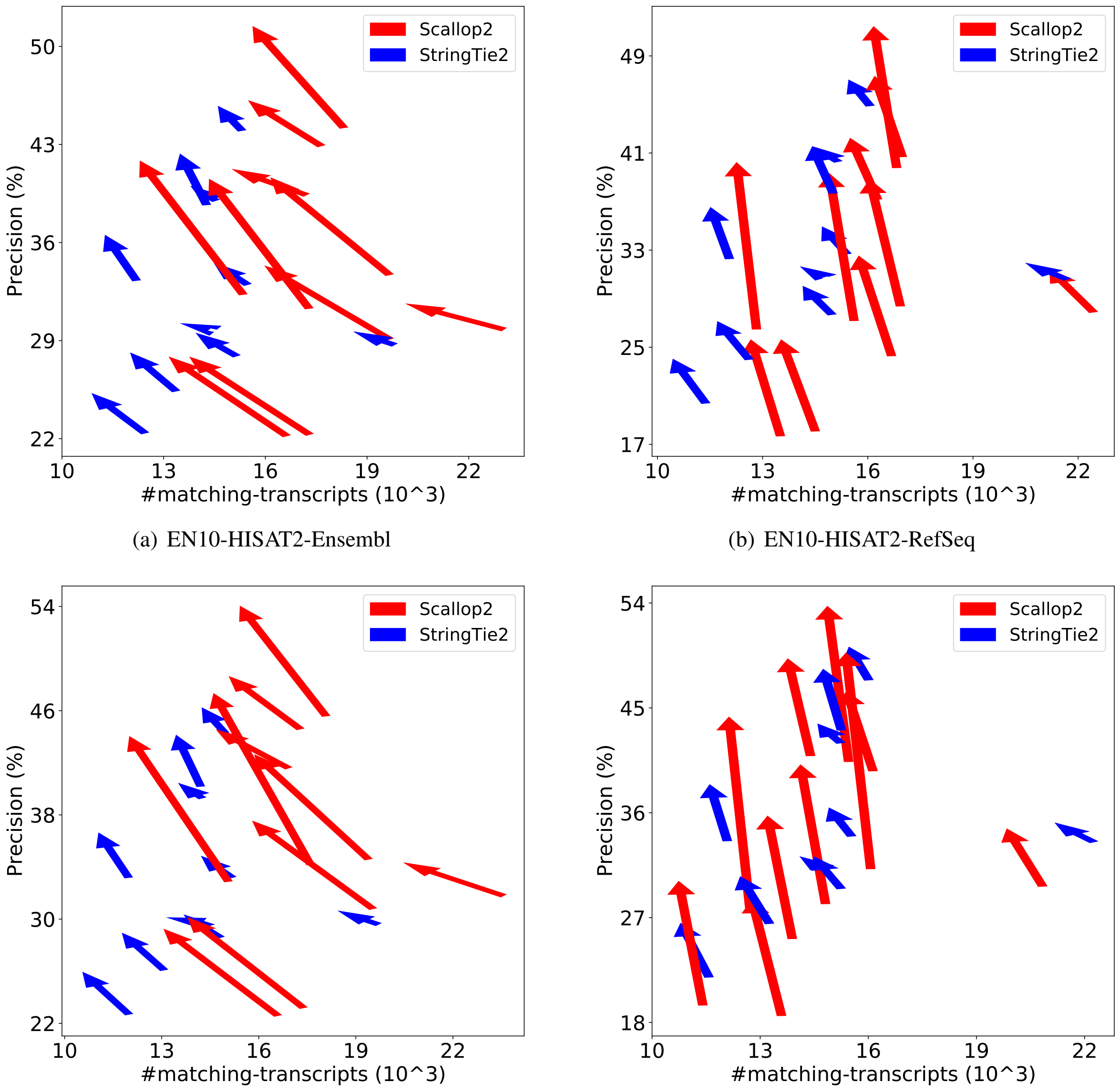

(a) EN10-HISAT2-Ensembl

(b) EN10-HISAT2-RefSeq

(c) EN10-STAR-Ensembl

(d) EN10-STAR-RefSeq

Fig A: Comparison of assembly accuracy of StringTie2 and Scallop2 before and after filtering out transcripts with intron retention. Each arrow represents a sample, pointing from the accuracy before filtering to that after filtering. The subfigures correspond to the 4 combinations of aligner (HISAT2 or STAR) and annotations (RefSeq or Ensembl) tested on EN10; both annotations are from GRCh38 genome build.

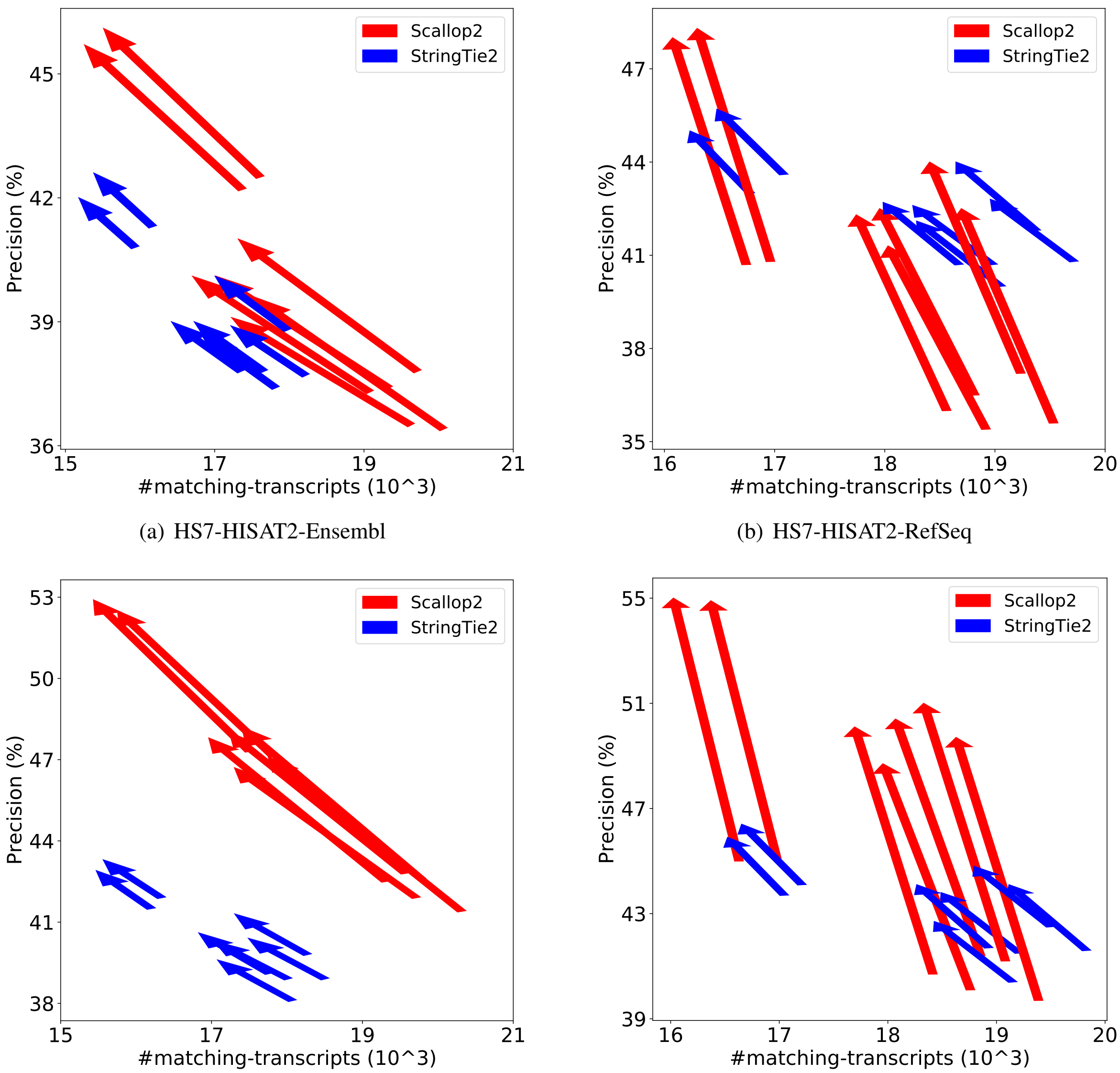

(a) HS7-HISAT2-Ensembl

(b) HS7-HISAT2-RefSeq

(c) HS7-STAR-Ensembl

(d) HS7-STAR-RefSeq

Fig B: Comparison of assembly accuracy of StringTie2 and Scallop2 before and after filtering out transcripts with intron retention. Each arrow represents a sample, pointing from the accuracy before filtering to that after filtering. The subfigures correspond to the 4 combinations of aligner (HISAT2 or STAR) and annotations (RefSeq or Ensembl) tested on HS7; both annotations are from GRCh38 genome build.

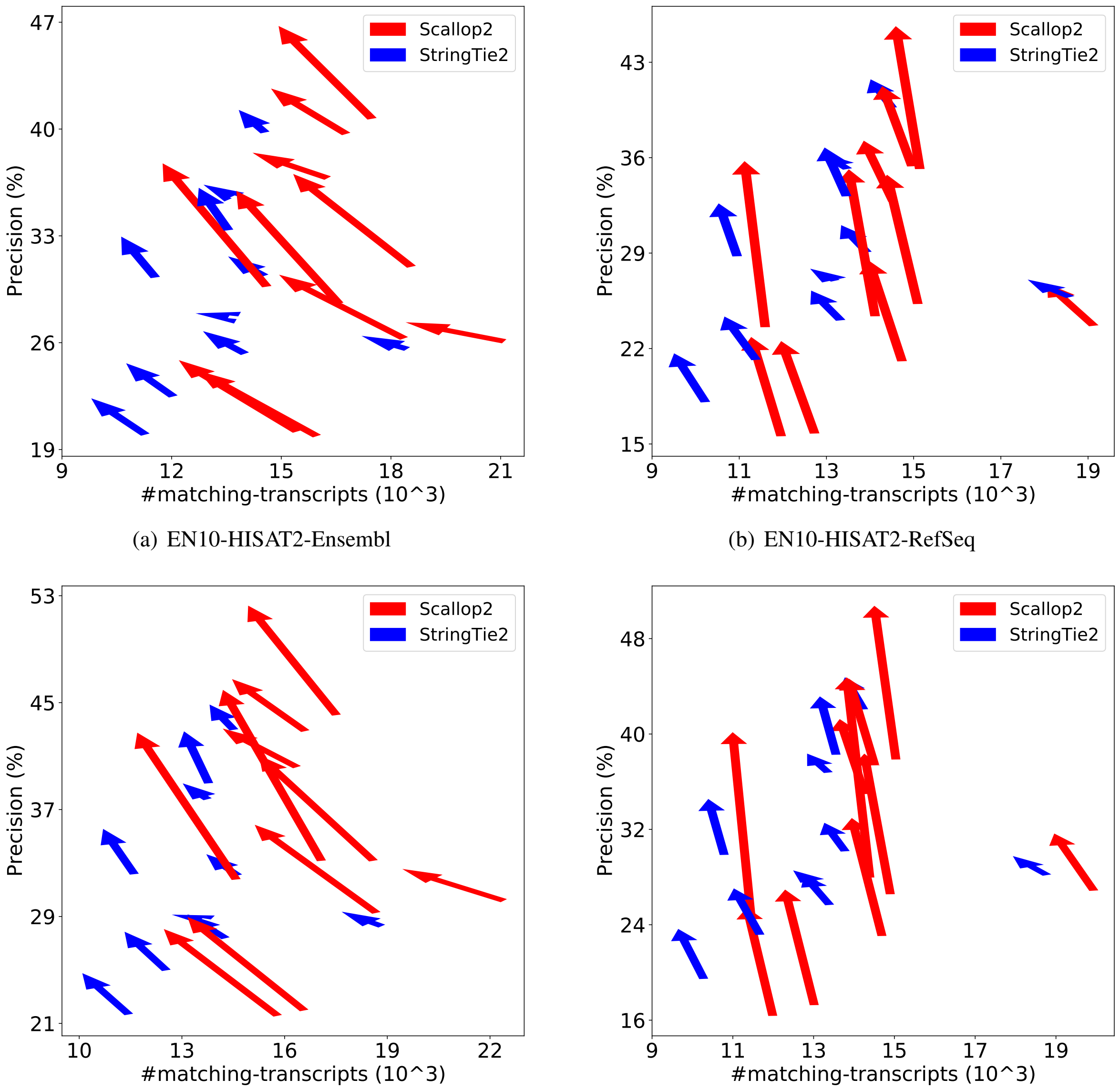

(a) EN10-HISAT2-Ensembl (b) EN10-HISAT2-RefSeq

(c) EN10-STAR-Ensembl (d) EN10-STAR-RefSeq

Fig C: Comparison of assembly accuracy of StringTie2 and Scallop2 before and after filtering out transcripts with intron retention. Each arrow represents a sample, pointing from the accuracy before filtering to that after filtering. The subfigures correspond to the 4 combinations of aligner (HISAT2 or STAR) and annotations (RefSeq or Ensembl) tested on EN10; both annotations are from T2T-CHM13 genome build.

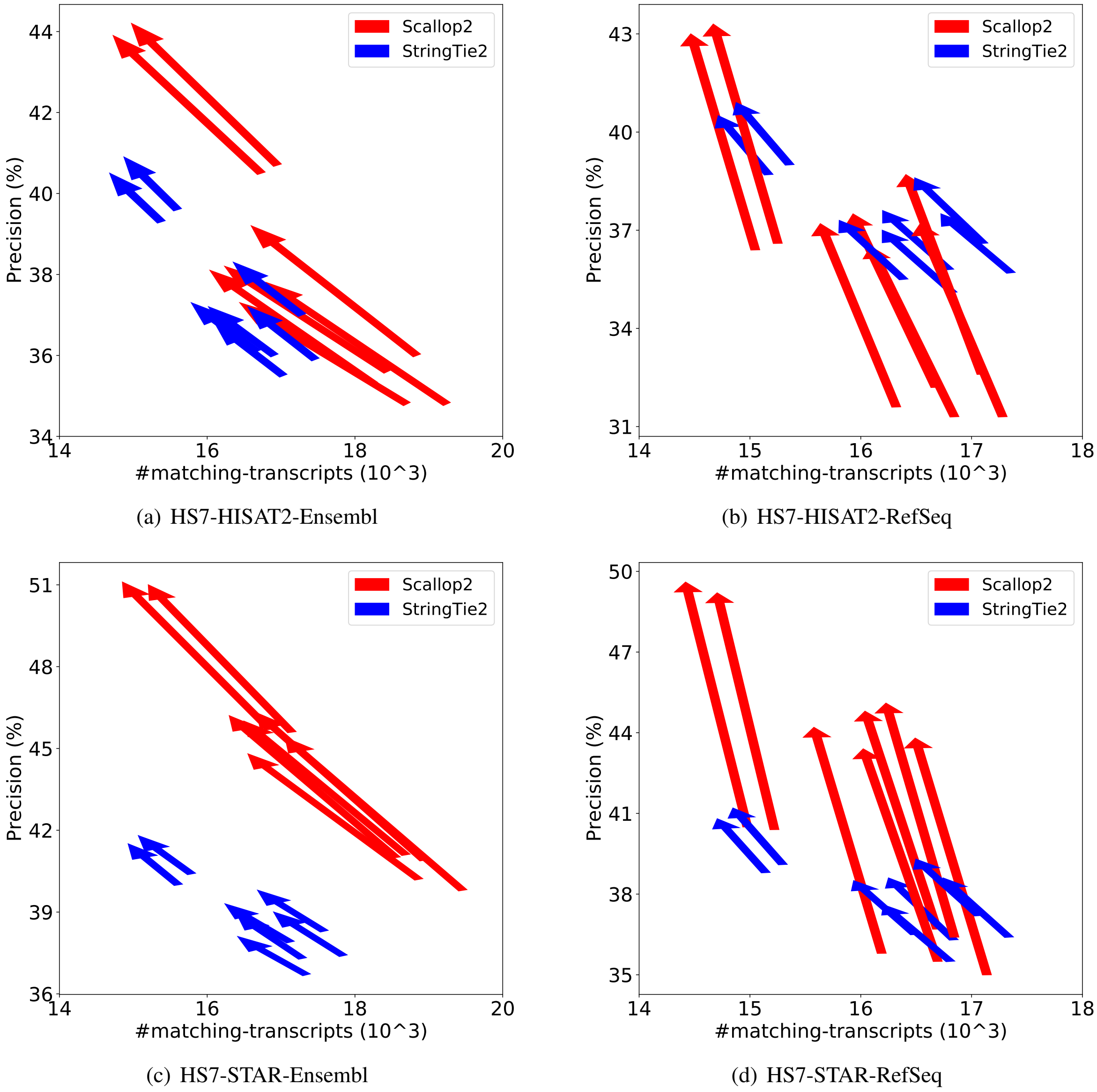

(a) HS7-HISAT2-Ensembl (b) HS7-HISAT2-RefSeq (c) HS7-STAR-Ensembl (d) HS7-STAR-RefSeq

Fig D: Comparison of assembly accuracy of StringTie2 and Scallop2 before and after filtering out transcripts with intron retention. Each arrow represents a sample, pointing from the accuracy before filtering to that after filtering. The subfigures correspond to the 4 combinations of aligner (HISAT2 or STAR) and annotations (RefSeq or Ensembl) tested on HS7; both annotations are from T2T-CHM13 genome build.

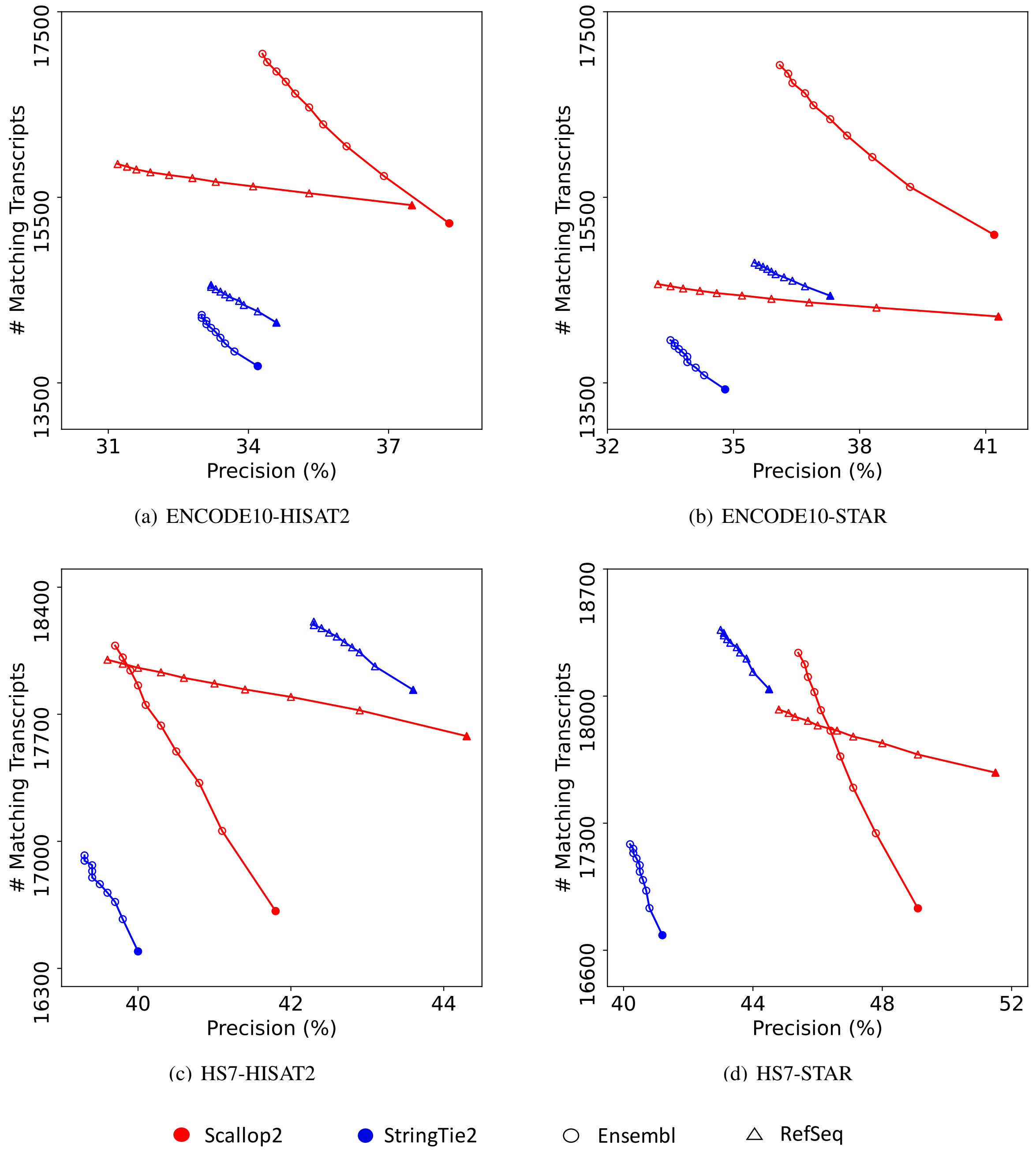

Fig E: Comparison of assembly accuracy of StringTie2 and Scallop2 after filtering out transcripts with intron retention with different setting of length ratio parameter. We vary length ratio parameter with a fixed coverage ratio parameter as 0.5, and report the average accuracy over all samples in each alignments. Each curve connects 10 points, corresponding to 10 different length ratio thresholds 0, 0.1, 0.2, 0.3, 0.4, 0.5, 0.6, 0.7, 0.8, 0.9. The default length threshold in irtool (0) is represented as a filled point. The assembly accuracy is evaluated on genome build GRCh38. (a) Assembly accuracy on HISAT2 alignments of ENCODE10. (b) Assembly accuracy on STAR alignments of ENCODE10 dataset. (c) Assembly accuracy on HISAT2 alignments of HS7 dataset. (d) Assembly accuracy on STAR alignments of HS7 dataset.

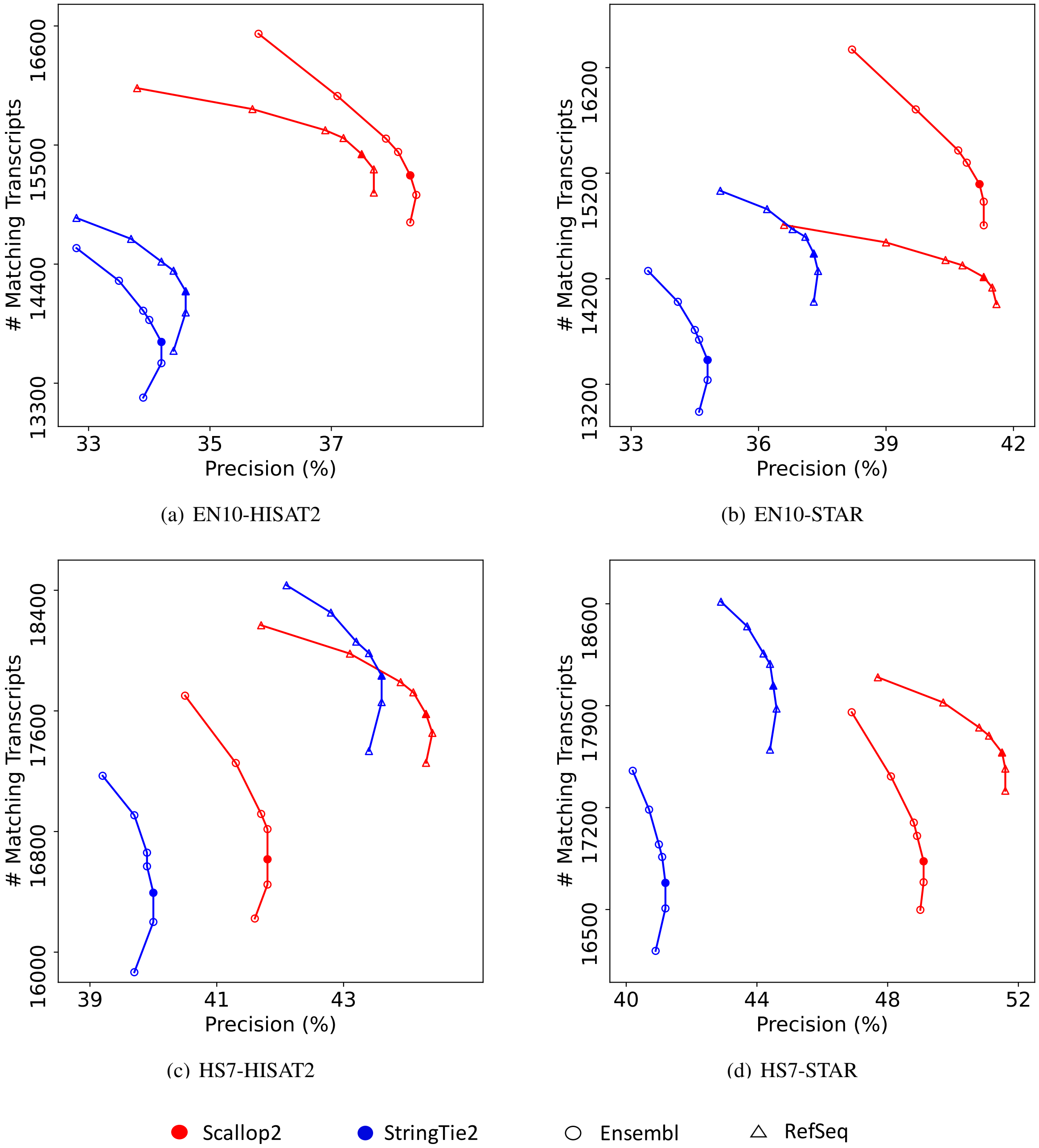

(a) EN10-HISAT2

(b) EN10-STAR

(c) HS7-HISAT2

(d) HS7-STAR

Fig F: Comparison of assembly accuracy of StringTie2 and Scallop2 after filtering out transcripts with intron retention with different setting of coverage ratio parameter. We vary coverage ratio parameter with a fixed length ratio parameter as 0, and report the average accuracy over all samples in each alignments. Each curve connects 7 points, corresponding to 7 different coverage ratio thresholds 0.1, 0.3, 0.5, 0.8, 1, 2, 5. The default coverage threshold in irtool (0.5) is represented as a filled point. The assembly accuracy is evaluated on genome build GRCh38. (a) Assembly accuracy on HISAT2 alignments of ENCODE10. (b) Assembly accuracy on STAR alignments of ENCODE10 dataset. (c) Assembly accuracy on HISAT2 alignments of HS7 dataset. (d) Assembly accuracy on STAR alignments of HS7 dataset.

Table A: Comparison of the assembly accuracy, measured with precision (%) and number of matching transcripts, of StringTie2 and Scallop2-ft using different annotations. In each combination (of dataset, aligner, genome build, annotation) the two metrics are averaged over all samples in the dataset. Symbol ⟨⟩ indicates that one method gets higher on one metric but lower on the other; symbol > indicates that StringTie2 outperforms on both metrics, while < indicates Scallop2 outperforms on both metrics. The three columns of *raw counts* give the number of samples in each category by comparing raw precision and recall. Samples in the ⟨⟩ category are further compared using the adjusted precision, and the number of samples are merged into either > or < category accordingly, shown in the two columns of under *adjusted*.

dataset	aligner	genome	annotation	StringTie2		Scallop2-ft		raw counts			adjusted	
				prec.	# mat.	prec.	# mat.	>	⟨⟩	<	>	<
EN10	HISAT2	GRCh38	RefSeq	32.1%	14906	37.4%	15421	0	2	8	1	9
EN10	HISAT2	T2T	RefSeq	28.2%	13298	33.0%	13705	0	1	9	1	9
EN10	STAR	GRCh38	RefSeq	34.3%	15113	41.2%	14223	0	6	4	6	4
EN10	STAR	T2T	RefSeq	30.2%	13279	37.6%	13765	0	0	10	0	10
HS7	HISAT2	GRCh38	RefSeq	41.5%	18523	44.1%	17599	0	7	0	7	0
HS7	HISAT2	T2T	RefSeq	36.6%	16429	39.1%	15676	0	7	0	7	0
HS7	STAR	GRCh38	RefSeq	42.2%	18695	51.4%	17581	0	7	0	0	7
HS7	STAR	T2T	RefSeq	37.1%	16425	45.7%	15640	0	7	0	1	6
	Summary		RefSeq	35.3%	15834	41.2%	15451	0	37	31	23	45
EN10	HISAT2	GRCh38	Ensembl	32.2%	14684	38.3%	15220	0	0	10	0	10
EN10	HISAT2	T2T	Ensembl	29.1%	13707	34.5%	14331	0	0	10	0	10
EN10	STAR	GRCh38	Ensembl	32.7%	14412	41.2%	15096	0	0	10	0	10
EN10	STAR	T2T	Ensembl	31.5%	13885	39.7%	14518	0	0	10	0	10
HS7	HISAT2	GRCh38	Ensembl	38.8%	17304	41.7%	16653	0	7	0	7	0
HS7	HISAT2	T2T	Ensembl	37.1%	16619	39.8%	15975	0	7	0	7	0
HS7	STAR	GRCh38	Ensembl	39.7%	17594	49.0%	16835	0	7	0	0	7
HS7	STAR	T2T	Ensembl	38.3%	16939	47.3%	16162	0	7	0	0	7
	Summary		Ensembl	34.9%	15643	41.4%	15599	0	28	40	14	54

Table B: Comparison of the assembly accuracy, measured with precision (%) and number of matching transcripts, of StringTie2-ft and Scallop2-ft using different annotations. In each combination (of dataset, aligner, genome build, annotation) the two metrics are averaged over all samples in the dataset. Symbol $\langle\rangle$ indicates that one method gets higher on one metric but lower on the other; symbol $>$ indicates that StringTie2 outperforms on both metrics, while $<$ indicates Scallop2 outperforms on both metrics. The three columns of *raw counts* give the number of samples in each category by comparing raw precision and recall. Samples in the $\langle\rangle$ category are further compared using the adjusted precision, and the number of samples are merged into either $>$ or $<$ category accordingly, shown in the two columns of under *adjusted*.

dataset	aligner	genome	annotation	StringTie2-ft		Scallop2-ft		raw counts			adjusted	
				prec.	# mat.	prec.	# mat.	$>$	$\langle\rangle$	$<$	$>$	$<$
EN10	HISAT2	GRCh38	RefSeq	34.6%	14150	37.4%	15421	0	3	7	1	9
EN10	HISAT2	T2T	RefSeq	30.5%	12690	33.0%	13705	0	3	7	0	10
EN10	STAR	GRCh38	RefSeq	37.3%	14439	41.2%	14223	2	4	4	6	4
EN10	STAR	T2T	RefSeq	33.0%	12738	37.6%	13765	0	1	9	0	10
HS7	HISAT2	GRCh38	RefSeq	43.6%	17834	44.1%	17599	4	3	0	7	0
HS7	HISAT2	T2T	RefSeq	38.5%	15857	39.1%	15676	4	3	0	7	0
HS7	STAR	GRCh38	RefSeq	44.5%	18038	51.4%	17581	0	7	0	0	7
HS7	STAR	T2T	RefSeq	39.2%	15883	45.7%	15640	0	7	0	1	6
	Summary		RefSeq	37.7%	15204	41.2%	15451	10	31	27	22	46
EN10	HISAT2	GRCh38	Ensembl	34.2%	13681	38.3%	15220	0	2	8	0	10
EN10	HISAT2	T2T	Ensembl	30.6%	12724	34.5%	14331	0	2	8	0	10
EN10	STAR	GRCh38	Ensembl	34.8%	13430	41.2%	15096	0	0	10	0	10
EN10	STAR	T2T	Ensembl	33.6%	12955	39.7%	14518	0	0	10	0	10
HS7	HISAT2	GRCh38	Ensembl	40.0%	16394	41.7%	16653	0	0	7	0	7
HS7	HISAT2	T2T	Ensembl	38.3%	15767	39.8%	15975	0	0	7	0	7
HS7	STAR	GRCh38	Ensembl	41.2%	16683	49.0%	16835	0	1	6	0	7
HS7	STAR	T2T	Ensembl	39.8%	16092	47.3%	16162	0	5	2	0	7
	Summary		Ensembl	36.6%	14716	41.4%	15599	0	10	58	0	68

Table C: Comparison of relative change in assembly accuracy after filtering out transcripts with partial intron retentions and with entire intron retentions, evaluated with different annotations from genome build GRCh38 as the reference. Numbers are averaged over all samples in each dataset.

dataset	aligner	filter type	annotation	StringTie2		Scallop2	
				Δ prec.	Δ # mat.	Δ prec.	Δ # mat.
EN10	HISAT2	partial	RefSeq	+4.8%	-3.0%	+20.0%	-3.1%
EN10	STAR	partial	RefSeq	+5.7%	-2.6%	+23.1%	-2.7%
HS7	HISAT2	partial	RefSeq	+3.5%	-2.2%	+12.5%	-2.7%
HS7	STAR	partial	RefSeq	+4.0%	-1.9%	+14.8%	-2.1%
	Summary		RefSeq	+4.5%	-2.4%	+17.6%	-2.6%
EN10	HISAT2	entire	RefSeq	+3.0%	-2.2%	+7.5%	-2.3%
EN10	STAR	entire	RefSeq	+3.3%	-2.0%	+9.5%	-2.3%
HS7	HISAT2	entire	RefSeq	+1.7%	-1.6%	+5.3%	-2.0%
HS7	STAR	entire	RefSeq	+1.7%	-1.7%	+6.4%	-1.9%
	Summary		RefSeq	+2.4%	-1.9%	+7.2%	-2.1%
EN10	HISAT2	partial	Ensembl	+4.0%	-4.2%	+10.7%	-11.4%
EN10	STAR	partial	Ensembl	+4.2%	-4.1%	+12.7%	-11.4%
HS7	HISAT2	partial	Ensembl	+2.2%	-3.4%	+5.5%	-8.7%
HS7	STAR	partial	Ensembl	+2.7%	-3.2%	+7.9%	-8.1%
	Summary		Ensembl	+3.3%	-3.7%	+9.2%	-9.9%
EN10	HISAT2	entire	Ensembl	+2.2%	-2.9%	+4.3%	-5.8%
EN10	STAR	entire	Ensembl	+2.4%	-3.0%	+5.1%	-6.2%
HS7	HISAT2	entire	Ensembl	+1.2%	-2.1%	+2.6%	-4.4%
HS7	STAR	entire	Ensembl	+1.1%	-2.2%	+3.7%	-4.4%
	Summary		Ensembl	+1.7%	-2.6%	+3.9%	-5.2%

Table D: Comparison of the assembly accuracy, measured with precision (%) and the number of matching transcripts, of StringTie2, Scallop2, StringTie1, and StringTie2-G (annotation-guided StringTie2). In each combination (of dataset, aligner, genome build, and annotation) the two metrics are averaged over all samples in the dataset. For StringTie2-G, the same annotation is used as the guiding-annotation and as the evaluation annotation.

dataset	aligner	genome	annotation	StringTie2		Scallop2		StringTie1		StringTie2-G	
				prec.	# mat.	prec.	# mat.	prec.	# mat.	prec.	# mat.
EN10	HISAT2	GRCh38	RefSeq	32.1%	14906	28.8%	16256	26.3%	11993	58.9%	32759
EN10	HISAT2	T2T	RefSeq	28.2%	13298	25.3%	14396	24.0%	11051	50.5%	24906
EN10	STAR	GRCh38	RefSeq	34.3%	15113	30.1%	14929	27.7%	12086	60.8%	32568
EN10	STAR	T2T	RefSeq	30.2%	13279	27.4%	14416	25.3%	11039	52.2%	24333
HS7	HISAT2	GRCh38	RefSeq	41.5%	18523	37.5%	18395	36.9%	14175	62.8%	32394
HS7	HISAT2	T2T	RefSeq	36.6%	16429	33.1%	16358	33.9%	13156	55.6%	26286
HS7	STAR	GRCh38	RefSeq	42.2%	18695	41.9%	18300	37.9%	14580	64.4%	33277
HS7	STAR	T2T	RefSeq	37.1%	16425	37.2%	16250	35.1%	13471	56.8%	26282
	Summary		RefSeq	35.3%	15834	32.7%	16163	30.9%	12694	57.8%	29101
EN10	HISAT2	GRCh38	Ensembl	32.2%	14684	32.7%	18205	27.3%	12080	66.9%	42797
EN10	HISAT2	T2T	Ensembl	29.1%	13707	29.9%	17100	24.7%	11417	62.5%	40100
EN10	STAR	GRCh38	Ensembl	32.7%	14412	34.2%	18133	27.2%	11919	67.6%	42899
EN10	STAR	T2T	Ensembl	31.5%	13885	32.9%	17406	26.4%	11536	65.7%	40905
HS7	HISAT2	GRCh38	Ensembl	38.8%	17304	38.6%	18971	35.5%	13611	66.3%	37128
HS7	HISAT2	T2T	Ensembl	37.1%	16619	36.8%	18165	33.9%	13152	63.9%	35558
HS7	STAR	GRCh38	Ensembl	39.7%	17594	43.7%	19145	37.1%	14293	68.9%	39749
HS7	STAR	T2T	Ensembl	38.3%	16939	42.0%	18355	36.1%	13869	66.9%	38066
	Summary		Ensembl	34.9%	15643	36.4%	18185	31.0%	12735	66.1%	39650

Table E: Comparison of the assembly accuracy, measured with precision (%) and the number of matching transcripts, of StringTie2, Scallop2, StringTie1, and StringTie2-G using the intersection of RefSeq and Ensembl, and the union of RefSeq and Ensembl from genome build GRCh38 as references for evaluation. In each combination (of dataset, aligner, and annotation) the two metrics are averaged over all samples in the dataset. For StringTie2-G, the same annotation is used as the guiding-annotation and as the evaluation annotation.

dataset	aligner	annotation	StringTie2		Scallop2		StringTie1		StringTie2-G	
			prec.	# mat.	prec.	# mat.	prec.	# mat.	prec.	# mat.
EN10	HISAT2	intersection	27.2%	12341	24.2%	13317	24.0%	10560	45.5%	19793
EN10	STAR	intersection	27.7%	11010	25.2%	13196	23.9%	10386	45.9%	19508
HS7	HISAT2	intersection	33.5%	14904	30.5%	14974	32.1%	12308	49.5%	21462
HS7	STAR	intersection	34.1%	15066	34.4%	15058	33.2%	12765	50.7%	21759
Summary		intersection	30.6%	13330	28.5%	14136	28.3%	11505	47.9%	20631
EN10	HISAT2	union	38.7%	17734	38.4%	21402	31.2%	13857	73.4%	53498
EN10	STAR	union	39.2%	17344	39.9%	21227	31.0%	13603	74.1%	53478
HS7	HISAT2	union	46.9%	20910	45.5%	22390	40.2%	15464	73.4%	45445
HS7	STAR	union	47.9%	21200	51.1%	22414	41.8%	16093	75.8%	48760
Summary		union	43.2%	19297	43.7%	21858	36.1%	14754	74.2%	50295
