# Supplementary material for: Transcript assembly and annotations: Bias and adjustment
Source: PLoS Comput Biol. 2023 Dec 21;19(12):e1011734. doi: 10.1371/journal.pcbi.1011734 (PMC10769104; doi:10.1371/journal.pcbi.1011734)
Supplement: Attachment — Submitted filename: revision-letter.pdf [file pcbi.1011734.s003.pdf]

Dear Editor,

We express our sincere gratitude to the referees and the editor for their diligent review of our manuscript. We are grateful to the referees for acknowledging the strengths of our work and offering constructive comments. We have conscientiously addressed all concerns raised, resulting in substantial improvements to the manuscript. Enclosed please find our detailed point-by-point responses to the comments. The major modifications are included in the newly added Supplementary Materials. Revisions to the Main text of the manuscript are highlighted in blue in the markup version.

Sincerely,
Qimin Zhang and Mingfu Shao

Reviewer #1: In the manuscript "Transcript Assembly and Annotations: Bias and Adjustment", the authors investigated the influence of different transcript annotations (RefSeq and Ensembl) on evaluating the accuracy of transcriptome assembly tools. Based on the observation that using different annotations to benchmark the assembly tools can lead to conflicting outcomes, the authors first compared the structural similarity between different annotations, and then investigated the transcript biotypes in different annotations and revealed that the differences between annotations are mainly related to intron retentions. Then, the authors proposed a tool to adjust the biases in intron retention for the assembled transcripts and provided guidance on how to choose the suitable assembler for the specific requirements.

To some extent, the research done by the authors make sense, since no criteria exists for deciding which annotation should be used to evaluate the transcriptome assemblers for now. And, different groups generally used different annotations to benchmark the assembler, e.g., in StringTie paper, the combination of the three annotations RefSeq, UCSC, and Ensembl was used as the ground truth, StringTie2 paper used only the RefSeq annotation, Scallop and Scallop2(developed by the authors of this manuscript) paper used the Ensembl annotation, PsiClass[PMID: 31676772] paper used the GENCODE annotation, and TransMeta[PMID: 35858749] paper used both RefSeq and GENCODE annotations, etc.

Overall, it appears to be a manuscript with rational methodology design and carefully-performed evaluation, while I still have some concerns as follows.

1. During the development of an algorithm, a specific transcriptome annotation library was generally used to train the computational model, and training with different annotations would greatly affect the tendency of the final assembly. This may be an important cause to result in conflicting outcomes when evaluating the assemblers with different annotations. I suggest the authors analyze the phenomenon from the root cause, rather than just from the final assembled results.

> We discussed the potential methodological cause of the conflicting outcomes between StringTie2 and Scallop2 in Supplementary Note 2.

2. As stated by the authors, Scallop2 identified much more intron retained transcripts than StringTie2. It seems that StringTie2 removes some kind of the aligned reads from the alignment files generated by Hisat2 or Star. Please the authors make more analysis from the methodology of StringTie2 and Scallop2, and this may provide new ideas for the development of new assemblers. Moreover, will StringTie1 have a similar performance as StringTie2? Since a great difference between StringTie2 and StringTie1 is StringTie2's more aggressive strategy for identifying and removing spurious spliced alignments.

We included the comparison with StringTie1 V1.3.6 (latest version of StringTie1) and other assemblers in Supplementary Table 1 and discussed the methodological difference between StringTie2 and StringTie1 in Supplementary Note 3.

3. Will it be good strategies to use the union or intersection of different transcriptome annotations as the ground truth to benchmark the transcriptome assembly tools?

We performed additional experiments using the union and intersection of Ensembl and RefSeq as ground-truth for evaluation. The results were shown in Supplementary Table 2 and analyzed in Supplementary Note 4.

4. According to the author's investigation, the annotation-guided assembly way (taking as input an annotation library to guide the assembly procedure) looks like a good strategy. And, StringTie2 has such an option, while Scallop2 doesn't. Please the authors discuss this.

We have added the comparisons of StringTie2 run with annotation (-G option) with other assemblers; the results are illustrated in Supplementary Table 1 and the discussion is added in Supplementary Note 3.

5. The figure 3 and 4 are referred to before figure 1 and 2 in the manuscript, figure numbers should fit citation's order, please the authors rearrange.

Thank you for pointing this out. We have fixed this.

6. In page 24, section 4.3, "... form a pair if they share at least one intron-exon boundary, i.e., $J(g1) \cap J(g2)$ …" should be "…i.e., $B(g1) \cap B(g2)$..".

Thank you for pointing this out. We have fixed it in the manuscript.

Reviewer #2: In this manuscript, the authors explored and identified potential biases when evaluating transcriptome assembly methods. The authors compared Scallop2 and StringTie2, two popular reference-guided transcriptome assemblers, on ten RNA-seq data, using two sets of genome annotations from RefSeq and Ensembl on hg38 and CHM13, respectively. The authors observed that Scallop2 and StringTie2's accuracy comparisons were inconsistent when using RefSeq and Ensemble annotations. The authors identified that one of the main reasons could be due to the intron retention transcripts. After removing qualified intron retention transcripts, the authors found Scallop2 obtained consistently higher precision and sensitivity in both annotation systems. The evaluations in this manuscript are well-designed, and the findings are important for future transcriptome assembler method development and benchmarks. However, there are several concerns the authors shall

address before the manuscript can be published on PLOS Computational Biology.

1. Partial intron retention should be split into two categories. Based on Figure 13, criterion 1 and criterion 2 may correspond to alternative transcription start/end sites. Traditionally, only criterion 3 should be considered intron retention as an alternative splicing event. The authors shall compare Scallop2 and StringTie2 on criterion1/2 and criterion 3 separately, which may provide more insights into the behaviors of the two transcriptome assemblers and the differences between RefSeq and Ensembl annotations.

> Thank you for the suggestion. We have added two options to irtool to enable the evaluation of criterion 1/2 and criterion 3 separately:
>
> - option -po to turn off partial intron retention and keep entire intron retention only, i.e. turn off criterion 1 and 2, keep criterion 3 only;
> - option -wo to turn off entire intron retention and keep partial intron retention only, i.e. turn off criterion 3, keep criterion 1 and 2 only.
>
> We have performed additional experiments of comparing Scallop2 and StringTie2 on criterion 1/2 and criterion 3 separately (Supplementary Table 5 and Supplementary Note 5).

2. For Figure 13's criterion1/2, except for the abundance ratio threshold, is there a length cutoff? For example, only when the exon of r covers more than 50% of the intron from another transcript t, r can be regarded as an intron retention transcript.

> Thank you for the suggestion. We have added an option -lr <double> into irtool to enable user customized length-ratio in criterion 1 and 2. We also performed additional experiments of turning the length-ratio parameter {0, 0.1, 0.2, 0.3, 0.4, 0.5, 0.6, 0.7, 0.8, 0.9}. The results have been added to Supplementary Figure 5 and discussed in Supplementary Note 6.

3. The default value for determining intron retention transcript is the p(r)/p(t)=0.5. Fold-2 change is a reasonable choice, but since one of the major findings in this manuscript is the differences in intron retention transcripts, the authors may need more justifications for this threshold. One suggestion is to draw precision-#matching-transcripts curves for StringTie2-ft and Scallop2-ft when varying the p(r)/p(t) threshold.

> Thank you for the suggestion. We have added an option -cr <double> into irtool to enable user customized coverage-ratio. We also performed additional experiments of turning the coverage-ratio parameter {0.1, 0.3, 0.5, 0.8, 1, 2, 5}. Please find details in Supplementary Figure 6 and Supplementary Note 7.

4. Even though the intron retention transcripts caused the evaluation discrepancy, ignoring them at all might be too drastic, as they could be real. The authors may consider using long reads or other evidence to confirm that intron retention transcripts are common in a sample. In other words, RefSeq annotation might be too conservative, and Ensembl annotation is a better representation of the transcripts, especially for intron retention transcripts. This might help establish a better guideline for future transcriptome assembler evaluations.

Thank you for the suggestion. We performed experiments of validating intron-retention transcripts annotated by Ensembl using two assemblies (produced by Bambu and StringTie2) from a long-reads dataset used in the LRGASP project. Please see the results and the discussion in Supplementary Note 8.

5. One minor concern is that the manuscript may have too many figures and tables. Though these figures are very informative, they may become distractions. For example, Figure 7-10 are large figure panels, and they are for underlying sample-level changes for Table 3. The authors may consider putting some of Figure 7-10 in supplementary figures to make the manuscript more concise.

Another example is Table 4, which is the comparison between the original StringTie2 output and the post-processed Scallop2-ft output. The information can be directly extracted from Table 1 and Table 5. Furthermore, comparing post-processed Scallop2-ft with an unprocessed StringTie2's would be deemed as an unfair comparison. Meanwhile, Table 5's comparison between StringTie2-ft and Scallop2-ft is a fairer comparison.

Thank you for the helpful suggestion. We have moved Figures 7-10 to Supplementary Figures 1-4, and moved Tables 4-5 to Supplementary Tables 3-4.
